# Moxibustion improves ovarian function based on the regulation of the androgen balance

**DOI:** 10.3892/etm.2022.11113

**Published:** 2022-01-04

**Authors:** Xun Jin, Jie Cheng, Jie Shen, Xing Lv, Qian Li, Yanyun Mu, Hua Bai, Yan Liu, Youbing Xia

Exp Ther Med 22:Article no. 1230, 2021; DOI: 10.3892/etm.2021.10664

Subsequently to the publication of the above article, the authors have realized that, in Fig. 2 on p. 4, the images selected for the A2 and the C2 data panels were inadvertently selected from the same original data source (note that the middle and right-hand columns in this figure were intending to show the x200 and x400 magnifications of the same data featured at a magnification of x20 in the left-hand column).

The authors have recaptured the x200- and x400-magnified pathological images through panoramic scanning of the original data. The revised version of Fig. 2, showing the new images captured for the A2-D2 and A3-D3 panels, is shown on the next page. Note that the error made in the original figure did not have a major impact on either the overall results or on the conclusions reported in this study. The authors thank the editor of *Experimental and Therapeutic Medicine* for allowing them the opportunity to publish this corrigendum, and apologize to the readership for any inconvenience caused.

## Figures and Tables

**Figure 2:**
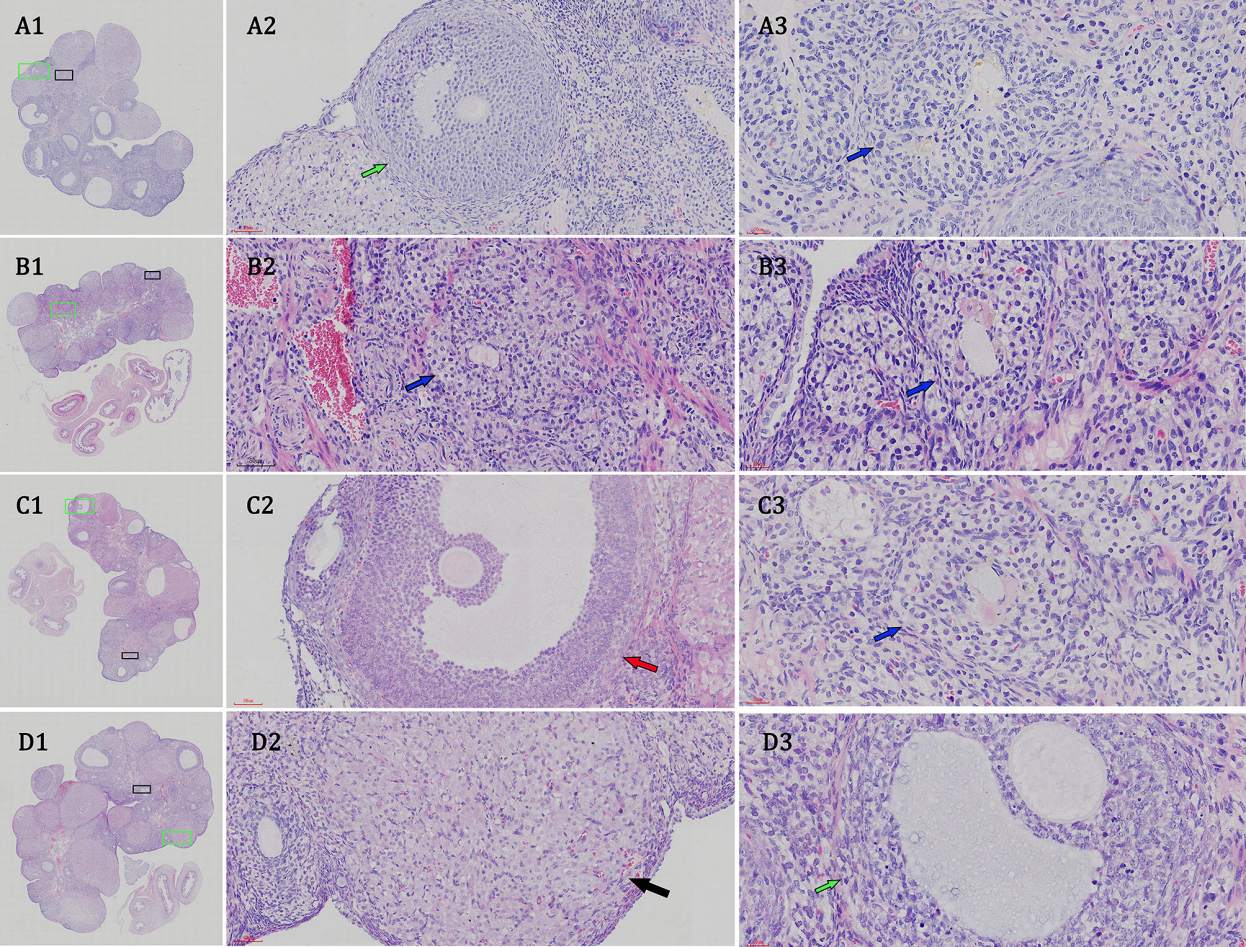
Moxibustion stimulation improves tripterygium glycoside-induced histopathological changes in rats. Histopathology images of ovaries from (A) the blank group, (B) model group, (C) moxibustion group 1 and (D) moxibustion group 2 (H&E staining; original magnification, x20, x200 and x400, in column 1, 2 and 3, respectively). The blue, red and black arrows indicate atretic follicles, mature follicular cells and the ovarian granuloma cells, respectively.

